# Metastasis of prostate carcinoma in the mandible manifesting as numb chin syndrome

**DOI:** 10.1186/1477-7819-12-401

**Published:** 2014-12-29

**Authors:** Secil Aksoy, Kaan Orhan, Sebnem Kursun, Mehmet Eray Kolsuz, Berkan Celikten

**Affiliations:** Department of Dentomaxillofacial Radiology, Faculty of Dentistry, Near East University, Dikmen Street, 90392 Lefkosa, Mersin 10, Turkey; Department of Dentomaxillofacial Radiology, Faculty of Dentistry, Ankara University, 06500 Besevler, Ankara, Turkey; Department of Endodontics, Faculty of Dentistry, Ankara University, 06500 Besevler, Ankara, Turkey

**Keywords:** distant metastasis, jaws, cone beam computed tomography, numb chin syndrome

## Abstract

**Background:**

Numb chin syndrome is an uncommon but well-recognized symptom in medical oncology. This condition can be related to metastatic neurological manifestation of malignancy, often with no clinically visible pathology. About 1% of oral cancers, which are located in the soft tissues and jaws, are metastases of primary tumors located elsewhere in the body. The posterior mandible is the most common site of metastasis of the oral region because of its rich blood supply in active areas of hematopoiesis. This article describes prostate carcinoma metastasis located in the mandible and temporomandibular joint of a 78-year-old male.

**Case presentation:**

A 78-year-old male patient presented to our outpatient clinic with a complaint of numbness and pain on the left site of the mandible. The patient stated that he had been suffering from this numbness for 1 to 2 months. In the medical anamnesis, it was discovered that patient had prostate carcinoma (CA) 5 years previous, and since then, he had visited his doctor periodically for an annual examination. In these examinations and on the basis of tests carried out at the hospital 1 year previous, it was stated that no CA relapse traces were detected. The patient had visited his dentist 2 months previous for pain and numbness of the left molar region.

**Conclusions:**

We report numb chin syndrome, which is an uncommon neurological manifestation of metastatic malignancy. The clinical course and rapid deterioration after the initial presentation of this syndrome is discussed. This clinical situation illustrates the importance of good medical history review prior to all procedures by the medical professions dealing with oncology patients. An awareness of this condition is crucial, especially in symptoms with unexplained facial pain and numbness.

## Background

Secondary malignancy of the jaws is uncommon, and when encountered, such cases may be diagnostically challenging. About 1% of oral cancers, which are located in the soft tissues and jaws, are metastases of primary tumors located elsewhere in the body [1].

The identification of these lesions, particularly soft tissue metastasis, can become a real diagnostic challenge because their clinical aspects can easily be mistaken for pyogenic granuloma, peripheral giant cell granuloma, or epulis [2]. The mandible is the most common site of metastasis of the oral region. The most common location is the molar region of the mandible, and the common primary lesions are located in the breast, adrenal gland, genital organs, thyroid gland, lung, prostate, and kidney [3]. Prostate CA prefers the jawbone because of its significant red marrow component as a metastatic target; for example, 11% of the jawbone metastases in men originated from the prostate gland compared with 1.5% in those metastases found in soft tissues [4].

The differentiation between primary tumor and metastasis is an important aspect. The histopathology of these lesions is very important. The appearance of oral metastatic lesions is a sign of advanced-stage malignant disease, with multiple metastases in other locations [5]. In some cases metastasis may develop in a recent post-extraction socket or even in the alveolar zone before extraction, giving rise to the indication of tooth removal [6]. The clinical presentation simulates common pathological conditions such as toothache, osteomyelitis, inflammatory hyperplasia, temporomandibular joint pain, trigeminal neuralgia, periodontal conditions, pyogenic or giant cell granuloma [6, 7], and accordingly, it may be difficult to diagnose such cases. Another clinical sign that is also usually seen, referred to in literature as ‘numb chin syndrome (NCS),’ occurs because of the involvement of the inferior alveolar branch of the mandibular nerve [7]. NCS is a sensory neuropathy that includes perineural spread of metastatic disease. Special attention should be given to patients with this syndrome, which should always raise the suspicion of a metastatic disease in the mandible [8].

The purpose of this report was to describe the clinical and radiological findings of numb chin syndrome as a manifestation a prostate carcinoma metastasis.

## Case presentation

**Figure 1:**
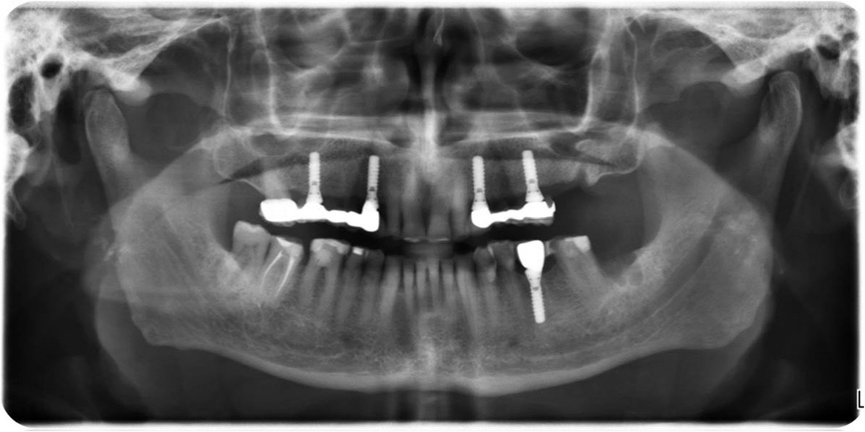
**Initial panoramic imaging taken by patient’s dentist 2 months ago and showing no apparent lesion.**

**Figure 2:**
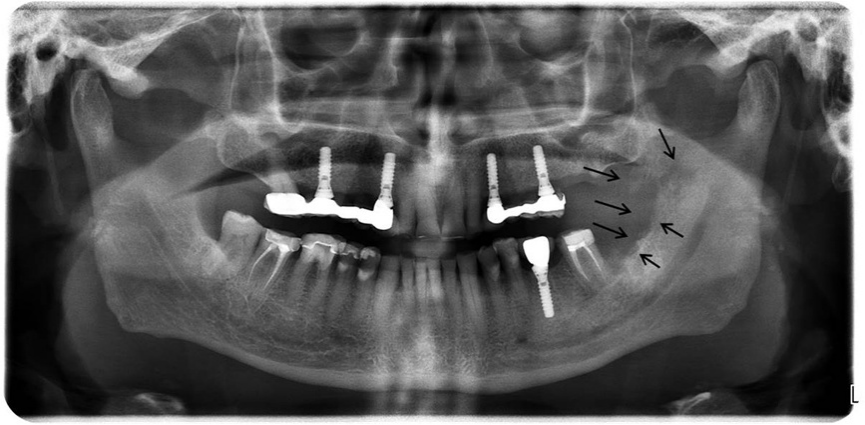
**Panoramic radiograph taken 1.5 months later and showing severe erosion on the left site of the mandible.**

**Figure 3:**
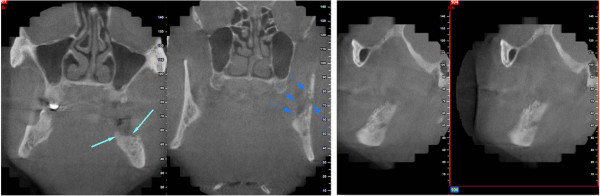
**Coronal and sagittal cone beam computed tomography (CBCT) images showing erosion in the molar region through the temporomandibular joint (TMJ) area.**

**Figure 4:**
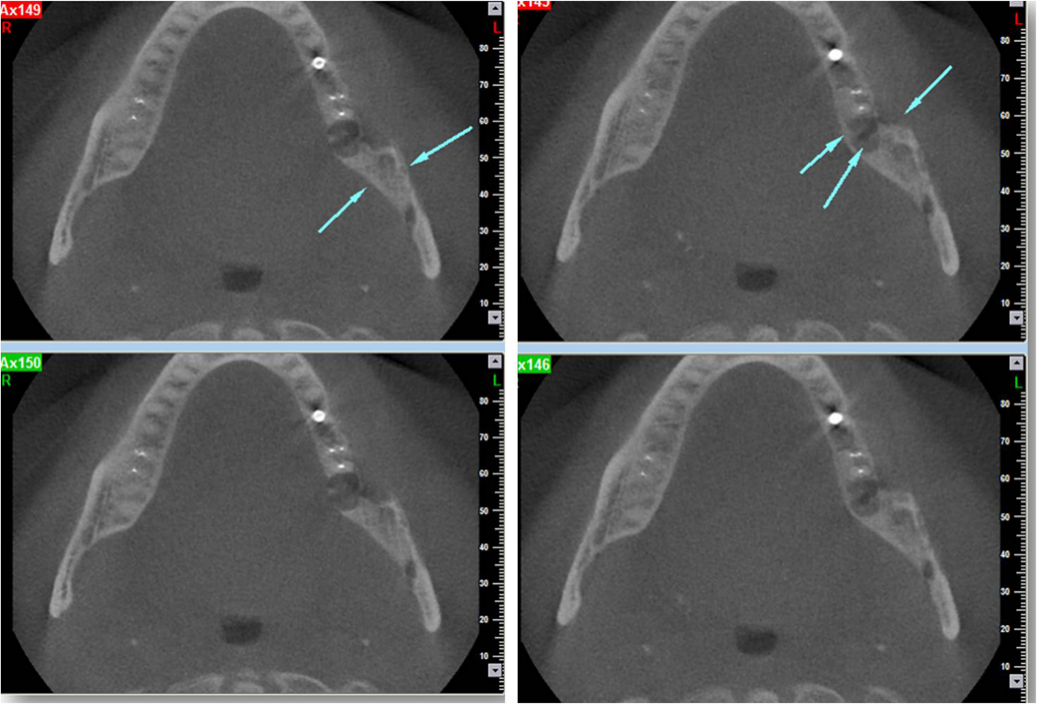
**Axial cone beam computed tomography (CBCT) images showing erosion in relation to the mandibular nerve on the level of the lingula mandible.**

**Figure 5:**
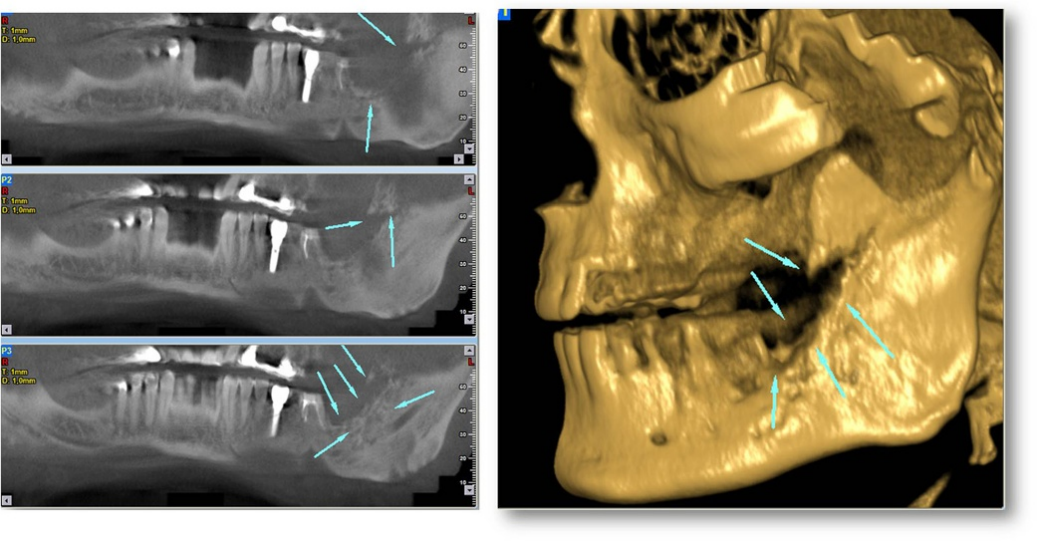
**Panoramic reconstructions and 3D cone beam computed tomography (CBCT) images showing severe moth-eaten shaped erosion in relation to the mandibular nerve.**

A 78-year-old male patient presented to our outpatient clinic with a complaint of numbness and pain on the left site of the mandible. The patient stated that he had been suffering from this numbness for 1to 2 months. In the medical anamnesis, it was discovered that patient suffered from prostate CA 5 years previous, and since then he had visited his doctor periodically for an annual examination. In the examinations and on the basis of tests carried out at the hospital 1 year previous, it was stated that no CA relapse traces were detected. The patient had visited his dentist 2 months previous for pain and numbness of the left molar region. He stated that after root canal treatment, his dentist extracted the second left molar due to calcification of the canal and also due to pain and numbness. After extraction, the numbness and pain of the patient worsened; two weeks later, a panoramic radiograph was taken by his dentist, who did not perform any treatment other than to advise follow-up (Figure 1).The patient presented to our clinic one and a half months later for further evaluation of his condition. Initially, a panoramic radiograph was taken. A moth-eaten shaped radiolucence was observed in the posterior mandible extending along the temporomandibular joint area. No pathologic findings were detected in other areas (Figure 2). After this initial appearance, in order to examine the lesion in detail, a decision was made to perform cone beam computed tomography (Newtom 3G, CBCT, QR Verona, Italy) with three-dimensional (3D) reconstruction to obtain a more precise location and definition of the pathologic features. In the 0.4 mm CBCT sections, a moth-eaten shaped radiolucent lesion extending from the molar region was observed. The lesion was also involved in the mandibular canal at the level of the lingula mandibula (Figures 3 and 4). Panoramic reconstructions and 3D CBCT images showed severe moth-eaten shaped erosion in relation to the mandibular nerve (Figure 5).

On consideration of the patient anamnesis and radiographs, a biopsy was planned for definitive diagnosis. The specimen showed that the bone marrow was filled with prostate cancer cells. The expression of prostate-specific antigen (PSA), cytokeratins (CKs) 7 and 20 was assessed by immunohistochemistry. The specimen showed a heterogeneous expression of PSA. CK stains of the specimen demonstrated a marker status of CK7 (-), and CK20 (-). Moreover, P504S/α-methylacyl coenzyme A racemase (AMACR) was used to as positive tissue marker to confirm the final diagnosis. A prostate carcinoma with metastasis was detected. The patient was sent for medical consultation. In the blood tests that were performed, gamma glutamine transferase was found to be over 295 iu/l, cea 817 ng/ml, and ca-15 600 U/ml. As a result, the patient was diagnosed as having recurrent prostate carcinoma, along with metastatic focuses that were detected at the femoral head, vertebra and shoulder head. No operation was planned because of severe metastasis of the CA throughout the body. No further follow-up was achieved. The patient died after 6 months due to the severe metastasis throughout his body.

## Discussion

Involvement of the jaws in CA metastasis occurs less frequently when compared to the involvement of other bones of the body [8]. Metastases to the jaws play a critical role in indicating the presence of a metastasis, a yet undiagnosed cancer, or recurrence of the disease [7], and they are important because of their poor diagnosis [3]. In 22% to 30% of cases, the oral presentation of metastasis is the first sign of malignant disease; in about 67% of cases, metastatic lesions are detected at the same time as the primary lesion [7]. The most common sites of primary tumors that lead to metastases of the jaws are breast (42%), adrenals (8.5%), genital organs (7.5%), and thyroid (6%) in women. The most common sites of primary tumors were lungs (22.3%), prostate (12%), kidney (10.3%), bone (9.2%) and adrenals (9.2%) in men [3]. The metastatic lesions of the maxillofacial region were located in the mandible, which is involved in 82% to 85% of such cases [9].

Mandibular metastases frequently occur in the premolar and molar areas. The clinical presentation of mandibular metastases can mimic clinical conditions such as toothache, temporomandibular joint pain, osteomyelitis, inflammatory hyperplasia, periodontal conditions, and pyogenic or giant cell granuloma [6]. Symptoms associated with metastases are pain, swelling, presence of intraoral mass, teeth mobility, gingival irritation, halitosis, trismus and NCS [6, 7]. Hirshberg *et al*. [4] also stated that rapid swelling, pain and paresthesia can be cardinal symptoms of jaw metastases.

Evaluation of NCS in a patient with a known malignancy should include a thorough physical examination to rule out causes, followed by panoramic radiography, and for detailed examination, computed tomography or magnetic resonance imaging and a whole-body bone scan. Another suggested modality for diagnosing metastatic lesions in the jaws is scintigraphy [10]. During the progression, the radiographic presentation is usually a radiolucent area with hazy outline. These lesions can simulate an infected cyst with their irregular outlines or as osteomyelitis by a moth-eaten appearance, as seen in this case. Also metastasis from the prostate can present as an osteoblastic lesion, usually seen as a radiopaque or mixed radiopaque-radiolucent lesion [10].

As in our patient, prostatic carcinoma is another cause for metastatic disease [11]. The study of Clausen and Poulsen [12] indicates that prostatic carcinoma is the primary source of more than 6% of metastatic lesions of the mandible, and a review of the literature by Vrebos and co-workers [13], revealed that 5% of the malignant lesions metastatic to the jaws were from the prostate gland. In a study of Daley *et al*[14], which evaluated 38 cases of metastatic disease, prostate CA was found to be the most common primary site (21%) for oral metastases. van der Waal and colleagues reported similar rates of 12% prostatic cancers in 24 cases.

Histopathologicially, the bone metastases of unknown primary tumors can be difficult to distinguish, especially for poorly differentiated adenocarcinomas or carcinoma. In order to distinguish the primary site, monoclonal antibodies specific to CK subtypes can be used to classify tumors according to the site of origin. The two most common CK stains are CK7 and CK20, and the combination of CK7 and CK20 immunoprofiling has been helpful to identify primary tumor sites [15]. Usually, CK profiles of prostate adenocarcinoma are negative for both CK7 and CK20. In order to have more accurate diagnosis, a recently developed molecular marker, such as AMACR, can be considered to confirm the primary site in cases of metastatic poorly differentiated adenocarcinoma, as seen in our case [16].

Reported cases of metastatic carcinoma of the prostate to the jaws illustrate the diversity of symptoms in affected patients including trismus, submandibular swelling, parotid swelling, preauricular swelling, periodontal abscess, sensory loss of branches of the trigeminal nerve, facial paralysis, eyelid ptosis and pathological fractures. In our case, similar symptoms (numbness and pain) were observed in the patient [8, 9, 12].

The majority of patients die some months after the appearance of an oral lesion. Nonetheless, even in cases of advanced malignant disease, palliative treatment is necessary for the control of pain, bleeding and impairment of chewing to improve the patient’s quality of life [17].

## Conclusions

This clinical situation illustrates the importance of good medical history review prior to all procedures by the medical professions dealing with oncology patients. Although there are few reports for carcinoma metastasis, medical professions should be aware of situations like this, and for early diagnosis, optimal clinical examination by means of radiographs and histopathology should be carried out. An awareness of this condition is crucial, especially in cases with symptoms of unexplained facial pain and numbness.

## Consent

Written informed consent was obtained from the patient for publication of this case report and any accompanying images. A copy of the written consent is available for review by the Editor-in-Chief of this journal.

## Abbreviations

AMACR: P504S/α-methylacyl coenzyme A racemase
CA: carcinoma
CBCT: cone beam computed tomography
CK: cytokeratin
NCS: numb chin syndrome
PSA: prostate-specific antigen.
